# Inter- and intralimb adaptations to a sensory perturbation during activation of the serotonin system after a low spinal cord transection in neonatal rats

**DOI:** 10.3389/fncir.2014.00080

**Published:** 2014-07-14

**Authors:** Misty M. Strain, Sierra D. Kauer, Tina Kao, Michele R. Brumley

**Affiliations:** ^1^Department of Psychology, Texas A&M UniversityCollege Station, TX, USA; ^2^Department of Psychology, Idaho State UniversityPocatello, ID, USA; ^3^Department of Psychology, City University of New YorkBrooklyn, NY, USA; ^4^Department of Psychology, New York UniversityNew York, NY, USA; ^5^Department of Neuroscience, Columbia UniversityNew York, NY, USA

**Keywords:** quipazine, stepping, range of motion, locomotion, development

## Abstract

Activation of the serotonin system has been shown to induce locomotor activity following a spinal cord transection. This study examines how the isolated spinal cord adapts to a sensory perturbation during activation of the serotonergic system. Real-time and persistent effects of a perturbation were examined in intact and spinal transected newborn rats. Rats received a spinal surgery (sham or low thoracic transection) on postnatal day 1 and were tested 9 days later. At test, subjects were treated with the serotonergic receptor agonist quipazine (3.0 mg/kg) to induce stepping behavior. Half of the subjects experienced range of motion (ROM) restriction during stepping, while the other half did not. Differences in stepping behavior (interlimb coordination) and limb trajectories (intralimb coordination) were found to occur in both intact and spinal subjects. Adaptations were seen in the forelimbs and hindlimbs. Also, real-time and persistent effects of ROM restriction (following removal of the perturbation) were seen in ROM-restricted subjects. This study demonstrates the sensitivity of the isolated spinal cord to sensory feedback in conjunction with serotonin modulation.

## Introduction

The role of serotonin (5-HT) in the activation and modulation of spinal locomotor circuits is well known, and has been demonstrated at the anatomical, neurophysiological, and behavioral levels of analysis. 5-HT receptors are found in high concentrations in locomotor regions of the spinal cord such as the cervical and lumbar enlargements (Jankowska et al., 1995, 1999), and bath application of 5-HT induces fictive locomotion in the spinal cord and spinal cord slices *in vitro* (e.g., Cowley and Schmidt, 1994; Garraway and Hochman, 2001; Hayes et al., 2008). During fictive locomotion induced by stimulation of the mesencephalic locomotor region, serotonergic boutons containing 5-HT_7_, 5-HT_2A_, and 5-HT_1A_ receptors were found to form synapses with or were in close proximity to activated lumbar motor neurons in the cat (Noga et al., 2009). *In vivo* behavioral studies further support the role of 5-HT in activating locomotor circuits. Treatment with 5-HT receptor agonists has been shown to produce alternated stepping behavior in intact and spinal transected fetal and newborn rats (McEwen et al., 1997; Brumley and Robinson, 2005; Brumley et al., 2012), adult rats (Kao et al., 2006), and adult mice (Lapointe and Guertin, 2008; Ung et al., 2008). Further, 5-HT receptor antagonists reduce locomotor activity produced by 5-HT treatment or electrical stimulation of the parapyramidal region (Cazalets et al., 1992; Liu and Jordan, 2005; Kao et al., 2006). Taken together, these studies point to an active role of the serotonergic system in modulating locomotor circuit activity.

Additionally, evidence suggests that sensory stimulation in conjunction with activation of 5-HT receptors increases locomotor function in spinal injured animals. For example, walking is improved in spinal injured mice following administration of the 5-HT_2A_ receptor agonist quipazine and robotic hindlimb training (Fong et al., 2005). Because 5-HT_2A_ receptor antagonists block quipazine-induced activity, quipazine is believed to act at the 5-HT_2A_ receptor (Ung et al., 2008). Quipazine and 8-OH-DPAT (a 5-HT_1A/7_ agonist) also facilitate stepping on a treadmill in a posture-dependent manner (Slawinska et al., 2012). Barbeau and Rossignol (1990) reported increases in step length and flexor and extensor responses to treadmill training in spinal cats following treatment with 5-HT substances. The degree to which such improvements in locomotor activity are due to activation of the 5-HT system, sensory stimulation, or both, is difficult to disentangle. However, it is important to understand how malleable these isolated or injured spinal circuits remain, particularly for the development of treatments aimed at functional recovery of locomotor behavior.

Sensory stimulation below the site of a spinal cord injury (SCI) is often used in rehabilitation efforts in individuals with SCI or other spinal disorders (i.e., spina bifida) where spinal sensorimotor systems have disrupted supraspinal regulation (Field-Fote, 2001; Pepin et al., 2003; Teulier et al., 2009). Treadmill training is a common form of sensory stimulation used to improve locomotor function after injury. The dramatic influence of sensory stimulation can be seen most clearly in research examining how animals adapt their behavior during and after exposure to stimulation. For example, studies examining trip responses on a treadmill or interlimb training have shown that training with these forms of sensory experiences can produce both real-time and persistent effects on limb activity and coordination (Pang et al., 2003; Brumley and Robinson, 2010; Zhong et al., 2012). Changes in response to sensory feedback also have been reported in an *in vitro* spinal cord-hindlimb preparation (Hayes et al., 2012). These studies point to the role of sensory feedback in modulating locomotor recovery.

Understanding how activation of spinal locomotor circuits, modulation of neural pathways, and sensory feedback interact may help shed light on why some rehabilitation strategies help and others seem to have no effect or even be harmful. The current study attempts to examine this complex interaction by investigating how the isolated spinal cord adapts to a sensory perturbation during activation of the 5-HT system in rats 9 days after a complete low thoracic spinal cord transection. In this study, rats received a spinal transection on postnatal day 1 (P1) and were tested on P10. During testing, stepping behavior was induced with the 5-HT agonist quipazine. Half of the rats received range of motion (ROM) restriction during stepping. By comparing interlimb and intralimb coordination in sham and spinal subjects, we examined the real-time and persistent effects of the sensory perturbation (ROM restriction) on quipazine-induced stepping behavior. We expected both sham and spinal subjects to show real-time and persistent responses to the sensory perturbation, as research has shown that neonatal transected rats retain considerable spinal plasticity (Weber and Stelzner, 1980; Stelzner et al., 1986). Thus, we expected subjects to keep their limbs more proximal to the body not only during ROM restriction, but also following removal of the perturbation.

## Materials and methods

### Subjects

Thirty-two Sprague–Dawley male rats received a low thoracic spinal cord transection or a sham operation on postnatal day 1 (P1; 24 h after birth) and were tested on P10. Adult rats were obtained from Simonsen Laboratories and mated in the PI's laboratory. Pregnant females were pair-housed until a couple days before birth, and then were housed individually. Animals were kept on a 12-h light:dark cycle with food and water available *ad libitum*. Animals were maintained in accordance with guidelines on animal care and use established by the NIH and Institutes of Laboratory Animal Resources (2011) and the Institutional Animal Care and Use Committee at Idaho State University.

### Study design

A total of six subjects were in each of the four groups. Subjects received spinal surgery (half received a complete low thoracic spinal transection; the other half underwent a sham spinal operation) on P1. To control for maternal behavior within each litter, all pups from the same litter received the same operation. Subjects were tested on P10: all subjects were treated with quipazine and either experienced ROM restriction or no ROM restriction. Each subject was tested in only one condition. Litters were culled to 6–8 pups on P1. Each subject within a group was selected from a different dam to avoid litter effects. Only males were used to avoid confounding group differences with sex effects.

### Spinal surgery

Spinal surgery was performed on P1. Subjects were voided before surgery commenced and showed evidence of recent feeding by the presence of a milk band across the abdomen. Subjects were anesthetized by hypothermia. The spinal transection technique used was that of Kao et al. (2006). Briefly, a partial laminectomy exposed the spinal cord from T8 to T10. For subjects that received a spinal cord transection, the spinal cord was physically cut at the T9–T10 level and a collagen matrix was injected into the transection site. Muscle and skin on the back was sutured. For subjects that received the sham operation, all procedures were the same except that the transection was not performed and no collagen matrix was injected.

Following surgery, subjects were administered a 50 μl subcutaneous injection of both Buprenex (0.1 ml of 0.04 mg/kg solution) and 0.9% (wt/vol) saline. Each subject was then placed with littermates and bedding from their home cage inside an infant incubator maintained at 35°C. When subjects recovered from anesthesia and looked healthy (i.e., their color was pink and behavior was normal), they were returned to the home cage with the dam. They remained with the dam until the day of testing (P10). To ensure that the dam would take care of her pups equally, pups from the same litter received the same type of spinal surgery (i.e., spinal transection or sham operation); litters were not mixed. Subjects were checked daily to ensure no complications or infections. Subcutaneous injections of saline were injected as needed on P5 to help with weight gain and hydration.

### Behavioral testing

Behavioral testing took place on P10. Subjects showed evidence of recent feeding. A minimum body weight cut-off of 15.50 g for inclusion in the study was used to ensure that subjects were within a fairly healthy weight range. However, comparison of body mass between the two surgery groups revealed a significant difference [*F*_(1, 22)_ = 26.54, *p* < 0.001], with spinal subjects (mean ± *SD*: 20.41 g ± 2.7) having lower body weights than sham subjects (26.93 g ± 3.4).

**Figure 1:**
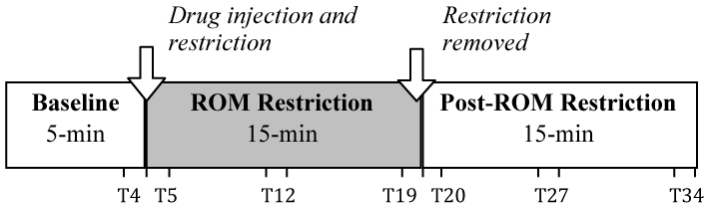
**Testing timeline.** After a 5-min baseline, subjects were injected with the serotonergic receptor agonist quipazine to induce stepping behavior. This was followed by a 15-min period of either ROM restriction (Plexiglas placed at 50% of maximum limb length) or no ROM restriction. Testing continued for a 15-min Post-ROM restriction period to look at the persistent effects of ROM restriction. One-minute time points beginning at T4, T5, T12, T19, T20, T27, and T34 were used to analyze the effects of ROM restriction on limb trajectories.

Subjects were individually tested inside an infant incubator that controlled temperature (30°C) and humidity. They were manually voided, and then acclimated to incubator conditions in a small plastic dish with up to two pups 30 min prior to testing. To start the test session, subjects were secured in the prone posture to a vinyl-coated horizontal bar using a jacket with adjustable straps across the neck and abdomen. The jacket did not impede limb movement; limbs hung pendantly in the air. Following a 5-min baseline, subjects were given an intraperitoneal injection of quipazine (3.0 mg/kg; Brumley et al., 2012) with volume of injection based on body weight (25 μl/5 g of body weight). Following injection, a 15-min ROM restriction period was imposed for half of the subjects. To induce ROM restriction (and thus alter cutaneous and proprioceptive feedback), a piece of Plexiglas was placed beneath the subject's limbs at 50% limb length when the limbs were fully extended. For the remaining half of the subjects, no ROM restriction was imposed. After the 15-min period of ROM restriction, the Plexiglas was removed and the subject was recorded for a 15-min post-ROM restriction period. In the no ROM restriction condition, subjects continued to be recorded for the 15-min post-ROM restriction period. Figure 1 shows the experimental timeline. The entire 35-min test session (5-min baseline, 15-min ROM restriction, and 15-min post-ROM restriction) was recorded using microcameras placed lateral and underneath the subject so that all limbs were visible. All sessions were recorded onto DVD for later behavioral scoring.

### Behavioral scoring

#### Interlimb coordination: stepping behavior

Stepping was scored during DVD playback using the underneath camera view with the software program JWatcher. Alternated and synchronized steps, along with non-stepping limb movements (e.g., twitches), were scored. Alternated steps were defined as occurring when the pup's homologous limbs exhibited sequential extension and flexion in one limb immediately followed by sequential extension and flexion in the other limb (Brumley et al., 2012). Synchronized steps were defined as occurring when the pup's homologous limbs exhibited simultaneous flexion and extension in both legs. Forelimbs and hindlimbs were scored in separate viewing passes. The scorer was blind to surgery condition. Intra- and interreliability for scoring was >90%.

#### Intralimb coordination: limb trajectories

**Figure 2:**
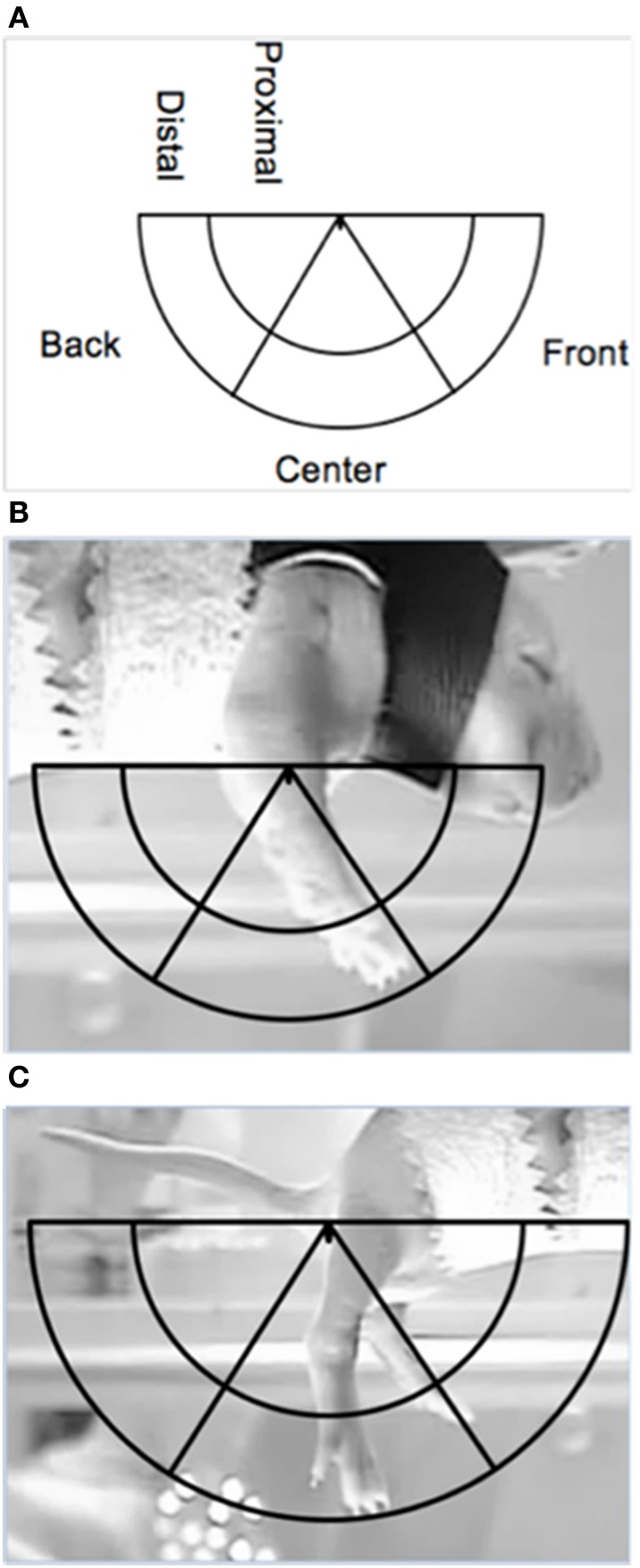
**Depiction of the limb trajectory space with the different limb trajectory areas labeled (A) along with illustrations of the placement of the trajectory area on proximal points for the (B) forelimbs and the (C) hindlimbs (the ventrum of the pup and the base of the tail, respectively)**.

Limb position was scored at specific time points to examine dorsal-ventral and rostral-caudal changes in the trajectory of limb movements, during DVD playback of the lateral camera view. The dorsal-ventral trajectory space was divided into a proximal and distal area, while the rostral-caudal trajectory space was divided into a front, center and back area (see Figure 2). These areas were calculated separately for the forelimbs and hindlimbs, for each subject.

The right forelimb and hindlimb were scored for each subject. Maximum limb length for each subject was determined from the baseline or post-ROM restriction period. To find maximum length, the ventrum of the subject and the tip of the toenail of the longest digit when the limb was fully extended were used as proximal and distal points on the forelimb, respectively. For the hindlimb, the base of the tail on the ventral side and the tip of the toenail of the longest digit when the limb was fully extended were used as proximal and distal points, respectively. Once the maximum length was found, a semicircle was made using the maximum length as the radius, with the center of the circle being placed on the appropriate proximal point (i.e., ventrum for forelimbs or base of tail for hindlimbs). An inner semicircle was then drawn within the aforementioned semicircle, with a radius equal to two-thirds the maximum length to create two dorsal-ventral limb trajectory areas: proximal (2/3 the distance of the maximum extension length) and distal (outer 1/3 the distance of the maximum extension length). To calculate the rostral-caudal trajectory areas, two lines were drawn from the appropriate proximal point (i.e., ventrum or base of tail) at 60- and 120-degrees of the entire 180-degree semicircle, thus creating a front (0–60 degrees), center (60–120 degrees), and back (120–180 degrees) trajectory area. Figure 2 depicts the trajectory areas and their placement on the forelimb and hindlimb proximal points. Due to the time-consuming nature of this analysis and preplanned comparisons, specific 1-min sections were scored: the last minute of baseline (T4), beginning of ROM restriction (T5), middle of ROM restriction (T12), end of ROM restriction (T19), beginning of the post-ROM restriction period (T20), middle of post-ROM restriction (T27), and end of the post-ROM restriction (T34) (see Figure 1). The scorer was blind to surgery condition.

### Histology

After testing, subjects were euthanized and preserved in 10% (wt/vol) formalin. Specimens were later examined under low magnification to verify complete spinal cord separation between spinal segments T9 and T10 (for spinal transected subjects) or an intact spinal cord (for shams).

### Data analysis

Data were analyzed using SPSS statistical software. A significance level of *p* ≤ 0.05 was adopted. *Post-hoc* tests used Tukey's Honestly Significant Difference.

#### Stepping behavior

Forelimb and hindlimb stepping was compared during the 35-min test session (5-min baseline, 15-min ROM restriction, and 15-min post-ROM restriction period). Frequencies of alternated steps and synchronized steps, along with the percentage of alternated and synchronized steps (calculated as a function of total limb movements) were summarized into 5-min time bins. Data were analyzed using repeated measures ANOVA, with surgery and ROM restriction condition as between subjects variables and time as a within subjects (repeated measure) variable. Forelimb and hindlimb data were analyzed separately.

#### Limb trajectories

Total time in each trajectory area (proximal, distal, front, center, and back) per 1-min section (as described above) was scored for hindlimbs and forelimbs. Data were analyzed using repeated measures ANOVA, with surgery and ROM restriction condition as between subjects variables and time as the within subjects (repeated measure) variable. Preplanned comparisons examined differences between baseline trajectories (T4) and those seen at the beginning of ROM restriction (T5), the end of ROM restriction (T19), after removal of ROM restriction (T20), and end of post-ROM restriction (T34) (see Figure 1). Additionally, comparison of sections within the ROM restriction period (T5, T12, and T19) was performed to determine real-time changes due to the perturbation. The time bin preceding (T19) and following (T20) post-ROM restriction were compared to determine immediate adaptations to removal of the perturbation. Comparison of all time bins during the post-ROM restriction period (T20, T27, and T34) was conducted to look for lasting changes. Forelimb and hindlimb data were analyzed separately.

## Results

Because spinal and sham subjects differed in body weight, we examined the correlation between body weight and total forelimb and hindlimb movements. There was no correlation (*p* = n.s.) between body weight and fore- or hindlimb activity. Therefore, we did not examine the influence of body weight further.

### Interlimb coordination: stepping behavior

#### Forelimb stepping

**Figure 3:**
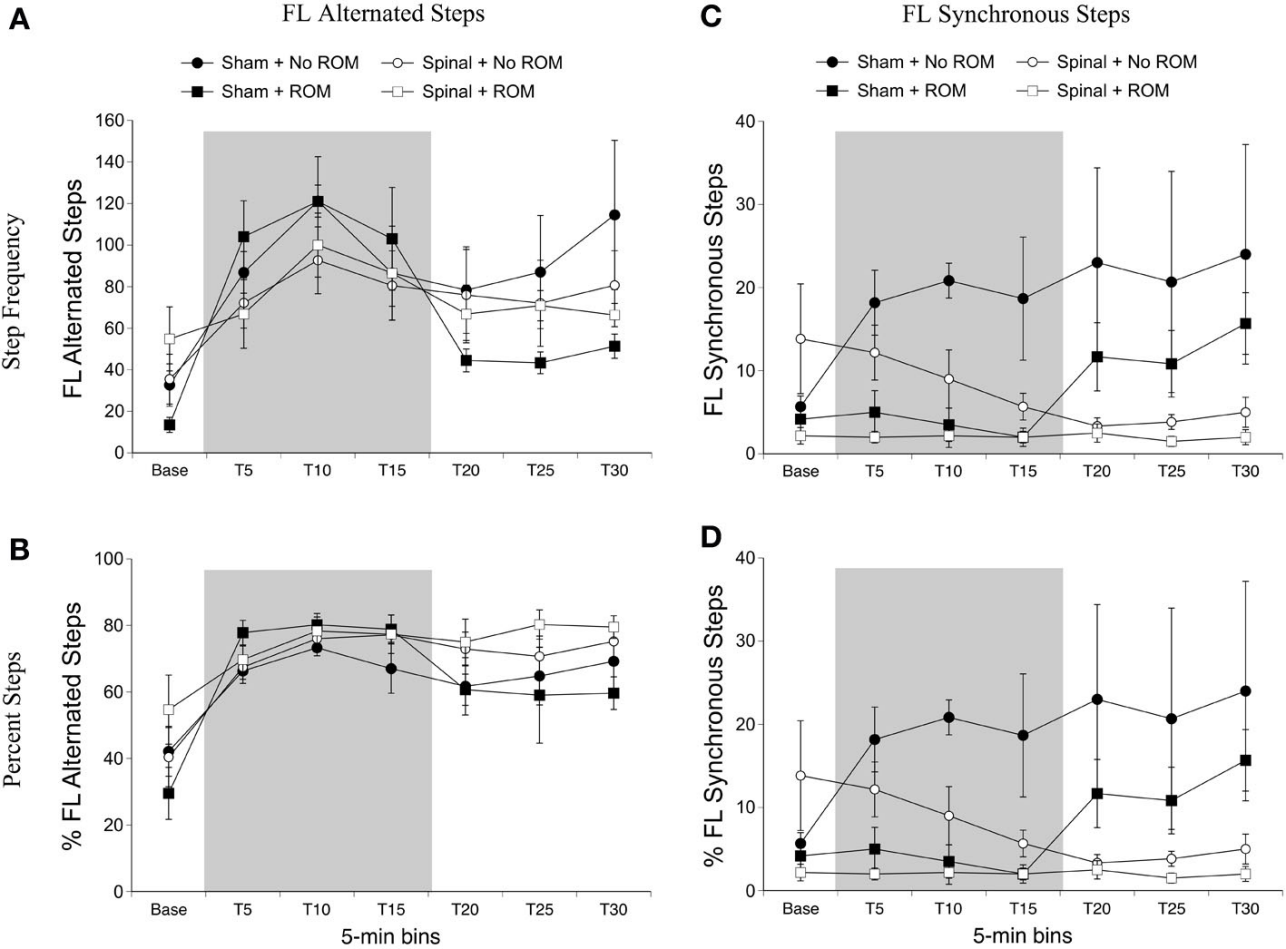
**Step frequency and percentage of forelimb steps for sham and spinal P10 rats by ROM condition.** Frequency **(A)** and percentage **(B)** of alternated forelimb steps, and frequency **(C)** and percentage **(D)** of synchronous forelimb steps are shown. The shaded gray region reflects the period of ROM restriction. Points show means; vertical lines are s.e.m.

***Alternated forelimb steps***. For frequency of alternated forelimb steps, there was a main effect of time [*F*_(6, 120)_ = 12.49, *p* < 0.001]. Follow-up analysis did not reveal any significant differences among time points; however, as shown in Figure 3A, there was a slight increase in forelimb steps after baseline followed by a reduction 15-min later. Because individual subjects may differ in their amount of total limb activity and thus the proportion of steps among subjects may vary, the percentage of alternated steps as a function of total movements (all steps + non-stepping movements) was examined. For percentage of alternated forelimb steps, there were main effects of surgery [*F*_(1, 20)_ = 6.10, *p* = 0.02] and time [*F*_(6, 120)_ = 17.73, *p* < 0.001]. The percentage of alternated forelimb steps significantly increased after baseline, and spinal subjects showed a significantly higher percentage of alternated steps compared to shams (Figure 3B).

***Synchronized forelimb steps***. For frequency of synchronized forelimb steps, there were effects of surgery [*F*_(1, 20)_ = 4.98, *p* = 0.04] and ROM restriction condition [*F*_(1, 20)_ = 4.98, *p* = 0.04], and a two-way interaction between surgery and time [*F*_(6, 120)_ = 3.55, *p* = 0.002]. As shown in Figure 3C, sham subjects expressed significantly more synchronized steps compared to spinal subjects at T20 and T30, with T25 approaching significance (*p* = 0.07). Additionally, subjects that received no ROM restriction expressed significantly more synchronized steps compared to subjects that did receive ROM restriction. The results for percentage of synchronized forelimb steps are very similar to those for frequency. There were main effects of surgery [*F*_(1, 20)_ = 7.94, *p* = 0.01] and ROM restriction condition [*F*_(1, 20)_ = 5.80, *p* = 0.03], and a two-way interaction between surgery and time [*F*_(6, 120)_ = 4.41, *p* < 0.001]. As shown in Figure 3D, sham subjects showed a significantly higher percentage of synchronized steps during the last 15 min of the test session (T15–T30) compared to spinal subjects, and subjects that did not receive ROM restriction showed a significantly higher percentage of synchronized forelimb steps compared to ROM-restricted subjects.

#### Hindlimb stepping

**Figure 4:**
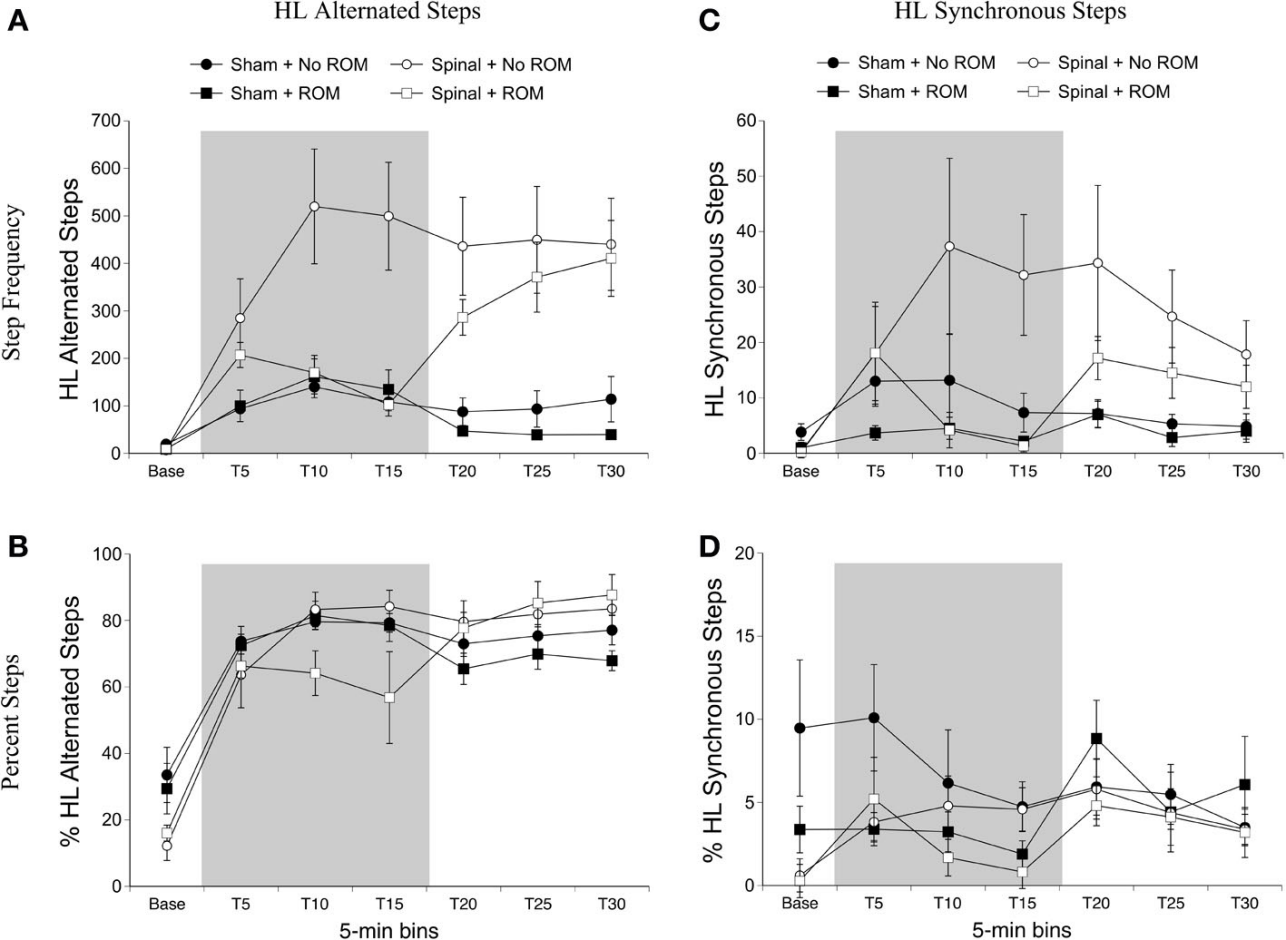
**Step frequency and percentage of hindlimb steps for sham and spinal P10 rats by ROM restriction condition.** Frequency **(A)** and percentage **(B)** of alternated hindlimb steps, and frequency **(C)** and percentage **(D)** of synchronous hindlimb steps are shown. The shaded gray region reflects the period of ROM restriction. Points show means; vertical lines are s.e.m.

***Alternated hindlimb steps***. For alternated hindlimb steps there were effects of surgery [*F*_(1, 20)_ = 21.77, *p* < 0.001] and time [*F*_(6, 120)_ = 20.89, *p* < 0.001], two-way interactions between surgery and time [*F*_(6, 120)_ = 11.51, *p* < 0.001] and ROM restriction condition and time [*F*_(6, 120)_ = 3.22, *p* = 0.006], and a three-way interaction between all factors [*F*_(6, 120)_ = 6.27, *p* < 0.001]. Follow-up analyses showed that significantly more alternated hindlimb steps occurred in spinal subjects compared to shams, and that alternated hindlimb stepping significantly increased after baseline (Figure 4A). To examine the three-way interaction, ROM restriction condition and time were examined within the two surgery conditions. As shown in Figure 4A, spinal subjects that experienced ROM restriction showed significantly fewer alternated steps at T10 and T15 compared to spinal subjects that did not experience ROM restriction. No significant differences were seen in shams. For percentage of alternated hindlimb steps there was a main effect of time [*F*_(6, 120)_ = 68.78, *p* < 0.001], a two-way interaction between surgery and time [*F*_(6, 120)_ = 6.06, *p* < 0.001], and a three-way interaction between surgery, ROM restriction condition and time [*F*_(6, 120)_ = 2.66, *p* = 0.02]. The percentage of alternated hindlimb steps increased after baseline and remained elevated throughout testing, and spinal subjects that received ROM restriction showed a significantly lower percentage of alternated steps at T10 compared to spinal subjects that did not experience ROM restriction (Figure 4B). Again we found no differences in sham subjects.

***Synchronized hindlimb steps***. For synchronized hindlimb steps there were main effects of surgery [*F*_(1, 20)_ = 5.36, *p* = 0.03] and time [*F*_(6, 120)_ = 5.57, *p* < 0.001], two-way interactions between surgery and time [*F*_(6, 120)_ = 2.47, *p* = 0.03], and ROM restriction condition and time [*F*_(6, 120)_ = 3.26, *p* = 0.005], and a three-way interaction between all factors [*F*_(6, 120)_ = 2.59, *p* = 0.02]. Significantly more synchronized steps occurred in spinal subjects compared to shams, and synchronized hindlimb stepping significantly increased after baseline (Figure 4C). To examine the three-way interaction, ROM condition and time were examined within the two surgery conditions. In spinal subjects, significantly fewer synchronized steps occurred at T15 for subjects receiving ROM restriction compared to those that did not receive restriction. There were no differences between ROM restriction conditions in the sham group. For percentage of synchronized hindlimb steps, there was a main effect of time [*F*_(6, 120)_ = 5.33, *p* < 0.001]. Follow-up analyses did not reveal any significant differences among the different time points. However, as can be seen in Figure 4D, most groups showed a slightly higher percentage of synchronized steps during the last half of the test session.

### Intralimb coordination: limb trajectories

#### Forelimb trajectories

**Figure 5:**
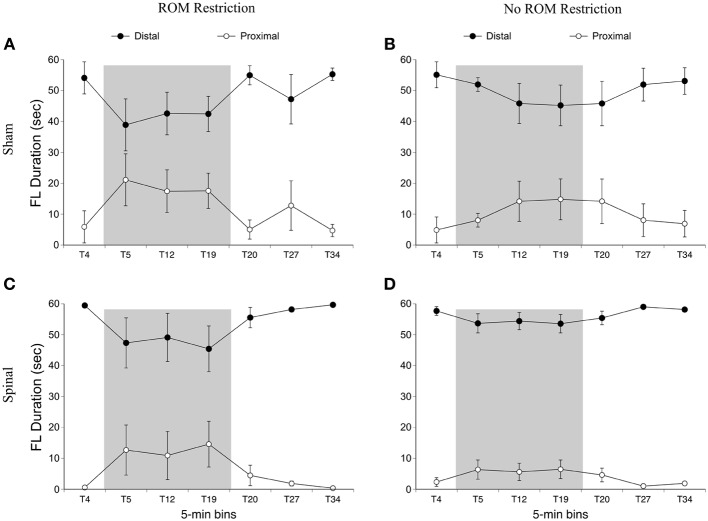
**Dorsal-ventral forelimb trajectories for sham and spinal P10 rats by ROM restriction condition.** Graphs show duration of time spent in distal and proximal trajectory areas for 1-min sections during baseline, the ROM restriction period, and the post-ROM restriction period for **(A)** sham subjects that received ROM restriction, **(B)** sham subjects that did not receive ROM restriction, **(C)** spinal subjects that received ROM restriction, and **(D)** spinal subjects that did not receive ROM restriction. The shaded gray region reflects the period of ROM restriction. Points show means; vertical lines are s.e.m.

We first examined time spent in the dorsal-ventral limb trajectory space. The *proximal* area was within 2/3 distance of maximum limb extension, whereas the *distal* area was the outer 1/3 distance (see Figure 2). We only analyzed time spent in one area (distal), as the pattern of effects are mirrored in the opposite (proximal) area. For time spent in the distal area, there was a main effect of time [*F*_(6, 120)_ = 3.94, *p* < 0.001]. Significantly less time was spent in the distal area for the forelimbs at T19 compared to baseline, and significantly more time was spent in the distal area at T34 compared to T19 (Figure 5).

**Figure 6:**
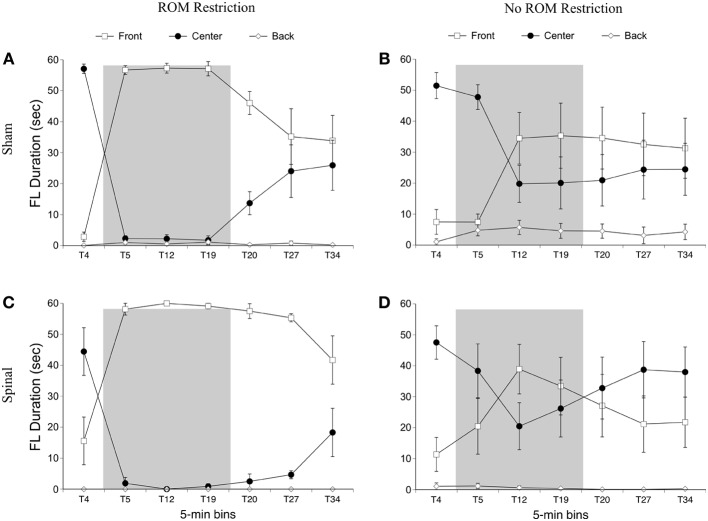
**Rostral-caudal forelimb trajectories for sham and spinal P10 rats by ROM restriction condition.** Graphs show duration of time spent in front, center, and back trajectory areas for 1-min sections during baseline, the ROM restriction period, and the post-ROM restriction period for **(A)** sham subjects that received ROM restriction, **(B)** sham subjects that did not receive ROM restriction, **(C)** spinal subjects that received ROM restriction, and **(D)** spinal subjects that did not receive ROM restriction. The shaded gray region reflects the period of ROM restriction. Points show means; vertical lines are s.e.m.

For the rostral-caudal limb trajectory space, we divided it into three equal areas: *front*, *center*, and *back* (see Figure 2). Time spent in each of these areas was analyzed separately. For time in the front area for the forelimbs there were effects of ROM restriction condition [*F*_(1, 20)_ = 14.52, *p* = 0.001] and time [*F*_(6, 120)_ = 28.86, *p* < 0.001], a two-way interaction between ROM restriction condition and time [*F*_(6, 120)_ = 7.67, *p* < 0.001], and a three-way interaction between all factors [*F*_(6, 120)_ = 2.24, *p* = 0.04]. ROM-restricted subjects spent significantly more time in the front compared to subjects that did not receive ROM restriction, and time in the front increased significantly after baseline. For ROM-restricted sham subjects, significantly more time was spent in the front at T5 and T12 and at T19 approached significance (*p* = 0.07), compared to sham subjects that did not receive ROM restriction. For ROM-restricted spinal subjects, significantly more time was spent in the front with the forelimbs from T5 to T27 compared to spinal subjects that did not receive ROM restriction. When surgery and time were examined within the two ROM restriction conditions, ROM-restricted spinal subjects showed significantly more time in the front at T20–27 than ROM-restricted shams. These effects can be seen in Figure 6.

For time in the center area with the forelimbs there were effects of ROM restriction condition [*F*_(1, 20)_ = 14.65, *p* = 0.001] and time [*F*_(6, 120)_ = 31.07, *p* < 0.001], a two-way interaction between ROM restriction condition and time [*F*_(6, 120)_ = 7.37, *p* < 0.001], and a three-way interaction between all factors [*F*_(6, 120)_ = 2.29, *p* = 0.04]. As shown in Figure 6, ROM-restricted subjects spent significantly less time in the center with their forelimbs compared to subjects that did not experience ROM restriction, and that time in center decreased after baseline. For ROM-restricted shams, significantly less time was spent in center at T5 and T12, with T19 approaching significance (*p* = 0.06), compared to shams that did not experience ROM restriction. For ROM-restricted spinal subjects, significantly less time was spent in center from T5 to T27 compared to spinal subjects that did not receive ROM restriction. Also, spinal subjects that experienced ROM restriction showed significantly less time in center at T20–27 than shams that received ROM restriction. Thus, both surgery groups decreased the amount of time in center with their forelimbs during the period of ROM restriction. Significant decreases in the center area also were seen in the forelimbs of spinal subjects during the first 10 min of post-ROM restriction (Figure 6).

For time in the back area with the forelimbs there were effects of surgery [*F*_(1, 20)_ = 4.61, *p* = 0.04] and ROM restriction condition [*F*_(1, 20)_ = 4.47, *p* = 0.05]. Sham subjects spent significantly more time in the back area compared to spinal subjects, and ROM-restricted subjects spent significantly less time in back compared to subjects that did not experience restriction (Figure 6).

***Summary***. No effects were seen for time spent in the distal area for the forelimbs as a result of ROM restriction. From T5 to T12, sham subjects that experienced ROM restriction decreased time in center and increased time in the front area. From T5 to T27, spinal subjects that experienced ROM restriction decreased time in center and increased time in the front area as well.

**Figure 7:**
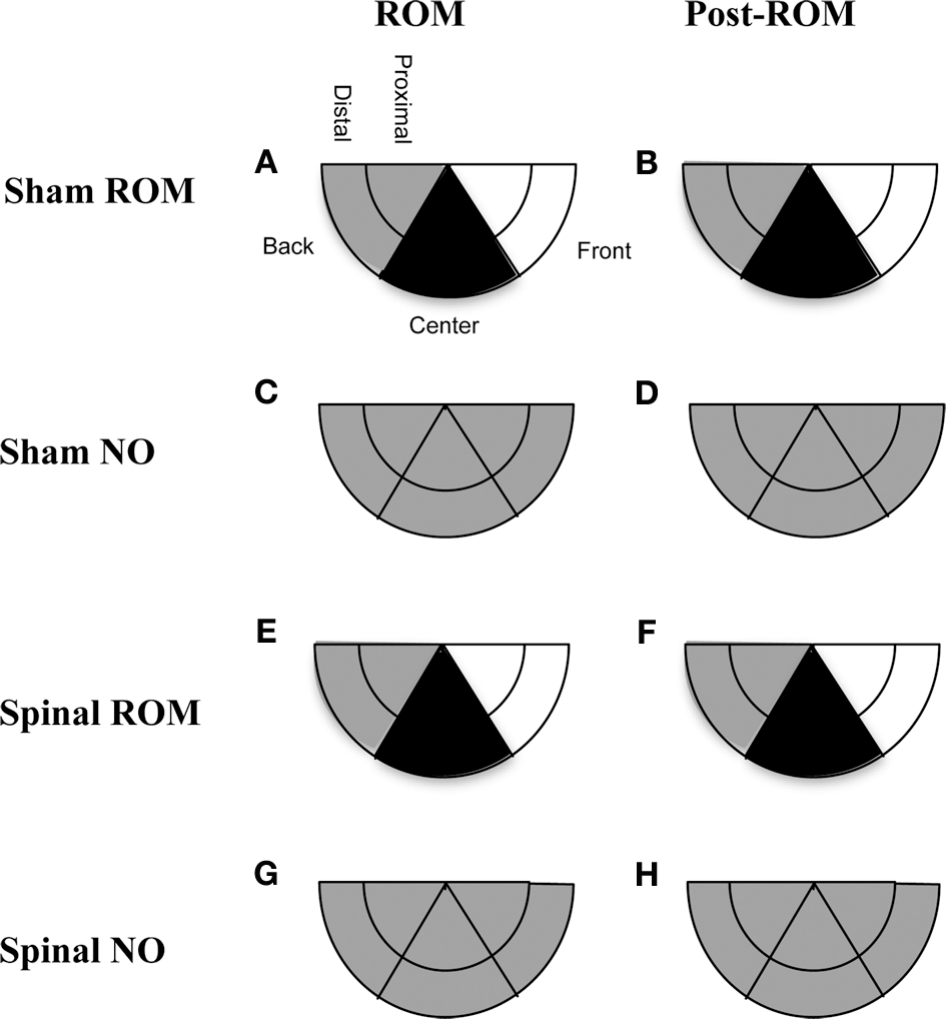
**Summary of changes in forelimb trajectories for sham and spinal P10 rats by ROM restriction condition.** Changes in limb trajectories are show for sham subjects that experienced ROM restriction **(A,B)**, shams that did not receive ROM restriction **(C,D)**, spinal subjects that received ROM restriction **(E,F)**, and spinal subjects that did not receive ROM restriction **(G,H)**. The shaded regions reflect an increase (white), maintenance (gray), or decrease (black) from baseline within that trajectory area during the designated time period.

***Preplanned comparisons***. For sham and spinal subjects that experienced ROM restriction, there were significant decreases in the center and significant increases in the front area with the forelimbs between baseline (T4) and the following time points: beginning of ROM restriction (T5), end of ROM restriction (T19), start of post-ROM restriction (T20), and end of post-ROM restriction (T34). These effects are summarized in Figure 7. Other comparisons were not significantly different between groups.

#### Hindlimb trajectories

**Figure 8:**
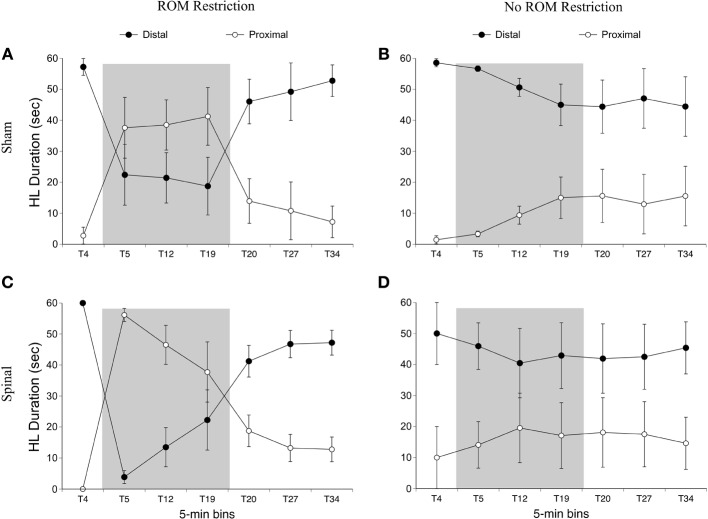
**Dorsal-ventral hindlimb trajectories for sham and spinal rats by ROM restriction condition.** Graphs show duration of time spent in distal and proximal trajectory areas for 1-min sections during baseline, ROM restriction, and the post-ROM restriction period for **(A)** sham subjects that received ROM restriction, **(B)** sham subjects that did not receive ROM restriction, **(C)** spinal subjects that received ROM restriction, and **(D)** spinal subjects that did not receive ROM restriction. The shaded gray region reflects the period of ROM restriction. Points show means; vertical lines are s.e.m.

For time in the distal area with the hindlimbs, there was an effect of time [*F*_(6, 120)_ = 19.12, *p* < 0.001] and an interaction between ROM restriction condition and time [*F*_(6, 120)_ = 17.22, *p* < 0.001]. For ROM-restricted subjects, significantly less time was spent in the distal area during ROM restriction (T5–T19), compared to subjects that did not experience ROM restriction. This can be seen in Figure 8.

**Figure 9:**
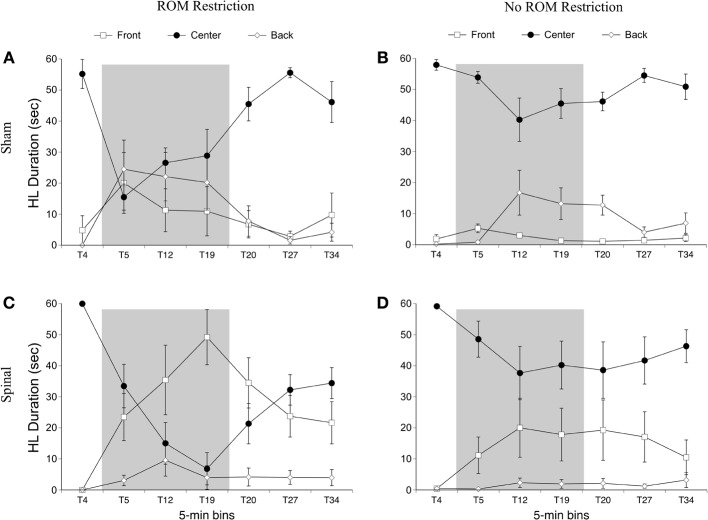
**Rostral-caudal hindlimb trajectories for sham and spinal P10 rats by ROM restriction condition.** Graphs show duration of time spent in front, center, and back trajectory areas for 1-min sections during baseline, ROM restriction, and the post-ROM restriction period for **(A)** sham subjects that received ROM restriction, **(B)** sham subjects that did not receive ROM restriction, **(C)** spinal subjects that received ROM restriction, and **(D)** spinal subjects that did not receive ROM restriction. The shaded gray region reflects the period of ROM restriction. Points show means; vertical lines are s.e.m.

For time in the front area there were effects of surgery [*F*_(1, 20)_ = 8.01, *p* = 0.01] and time [*F*_(6, 120)_ = 8.54, *p* < 0.001], and interactions between surgery and time [*F*_(6, 120)_ = 7.31, *p* < 0.001] and ROM restriction condition and time [*F*_(6, 120)_ = 2.44, *p* = 0.03]. Spinal subjects showed significantly more time in the front with their hindlimbs compared to shams, and significantly more time in the front after baseline (see Figure 9). Follow-up analysis of the two-way interaction between surgery and time revealed that spinal subjects spent significantly more time in the front from T12 to T27 compared to shams. Follow-up analysis of the interaction between ROM restriction condition and time showed that ROM-restricted subjects spent significantly more time in the front at T5 and T19 compared to subjects that did not receive ROM restriction.

For time in the center area with the hindlimbs there were effects of ROM restriction condition [*F*_(1, 20)_ = 10.28, *p* = 0.004] and time [*F*_(6, 120)_ = 23.79, *p* < 0.001], interactions between surgery and time [*F*_(6, 120)_ = 5.30, *p* < 0.001] and ROM restriction condition and time [*F*_(6, 120)_ = 6.32, *p* < 0.001], and a three-way interaction between all factors [*F*_(6, 120)_ = 3.05, *p* = 0.008; Figure 9]. ROM-restricted subjects spent significantly less time in center compared to subjects that did not experience restriction, and time in center significantly decreased after baseline. For shams that experienced ROM restriction, less time was spent in center at T5 compared to subjects that did not receive restriction. For spinal subjects, less time was spent in center at T19 and approached significance at T12 (*p* = 0.07), compared to subjects that did not receive ROM restriction. Also, for spinal subjects that experienced ROM restriction, less time was spent in center from T19 to T27 compared to shams that experienced ROM restriction. These effects can be seen in Figure 9.

For time in the back area with the hindlimbs, there were effects of surgery [*F*(6, 120) = 6.46, *p* = 0.02] and time [*F*(6, 120) = 7.10, *p* < 0.001], an interaction between surgery and time [*F*(6, 120) = 3.71, *p* = 0.002] and ROM restriction condition and time [*F*(6, 120) = 2.84, *p* = 0.01], and an interaction between all factors [*F*(6, 120) = 2.445, *p* = 0.04]. Shams spent significantly more time than spinal subjects in back with their hindlimbs, and time in back significantly increased after baseline. ROM-restricted shams showed significantly more time in back at T5 compared to shams that did not experience restriction. Also, ROM-restricted spinal subjects showed significantly more time in back at T5 compared to ROM-restricted shams. For subjects that did not experience restriction, spinal subjects showed significantly more time in the back area at T20 with T19 approaching significance (*p* = 0.06), compared to shams.

***Summary***. ROM-restricted subjects spent less time in the distal area with the hindlimbs during the restriction period. Additionally, for shams that experienced restriction, less time was spent in the center and more time was spent in the back at T5. For spinal subjects that experienced restriction, less time was spent in the center and more time was spent in the front at T19.

**Figure 10:**
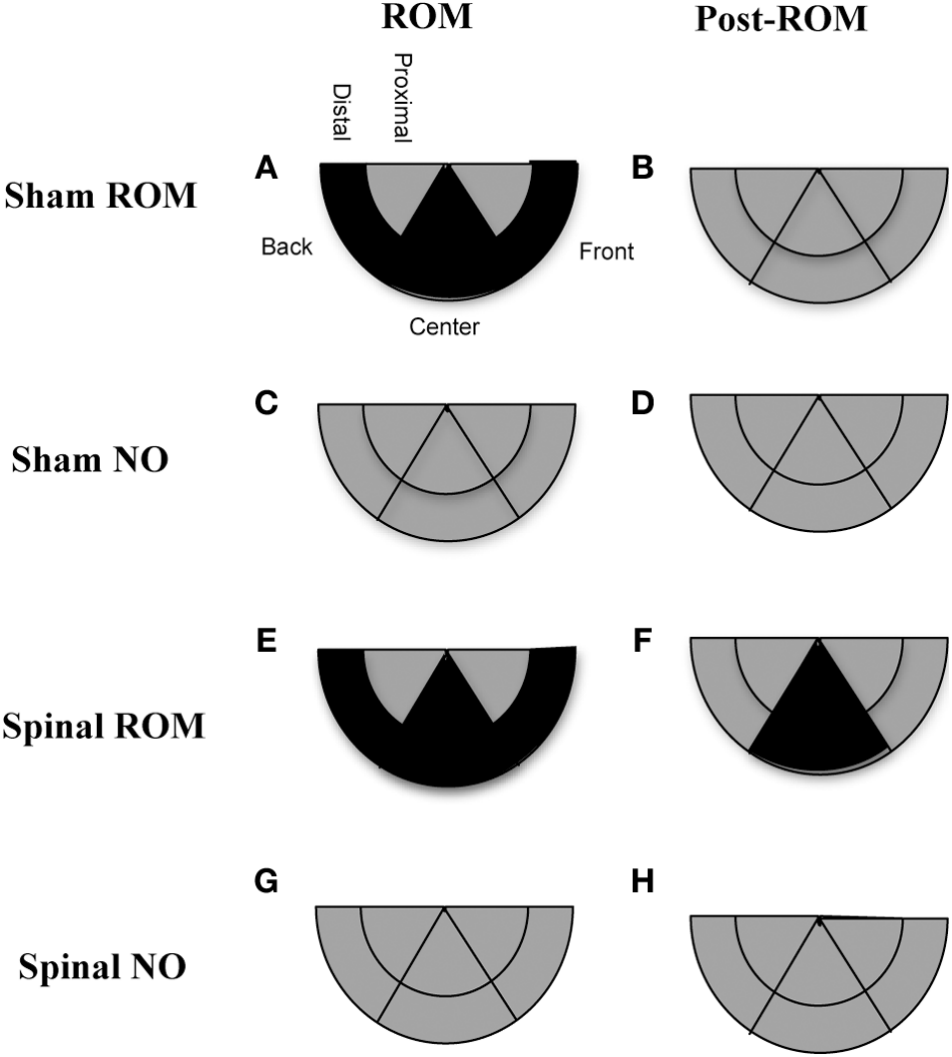
**Summary of changes in hindlimb trajectories for sham and spinal P10 rats by ROM restriction condition.** Changes in limb trajectories are show for sham subjects that experienced ROM restriction **(A,B)**, shams that did not receive ROM restriction **(C,D)**, spinal subjects that received ROM restriction **(E,F)**, and spinal subjects that did not receive ROM restriction **(G,H)**. The shaded regions reflect a maintenance (gray) or decrease (black) from baseline within that trajectory area during the designated time period.

***Preplanned comparisons***. For sham subjects that experienced ROM restriction, significantly less time was spent in the center and distal areas with the hindlimbs between baseline (T4) and beginning of ROM restriction (T5) and end of ROM restriction (T19). Other comparisons were not significant in shams. For spinal subjects that experienced ROM restriction, there were significant decreases for time spent in the distal area with the hindlimbs between the baseline (T4) and beginning and end of ROM restriction (T5 and T19). There were significant decreases in center between baseline (T4) and the following times: end of ROM restriction (T19), beginning of post-ROM restriction (T20), and end of post-ROM restriction (T34). There also was a significant decrease in center between the beginning and end of ROM restriction (T5 and T19). For sham and spinal subjects that did not receive ROM restriction, there were no significant differences within the different trajectory areas. These effects are summarized in Figure 10.

## Discussion

This study demonstrates the sensitivity of the isolated spinal cord to sensory (cutaneous and proprioceptive) feedback in conjunction with 5-HT modulation. The 5-HT_2A_ receptor agonist quipazine induced forelimb and hindlimb stepping in both sham and spinal transected P10 rats. However, only spinal subjects modulated their hindlimb stepping during ROM restriction. Both sham and spinal subjects showed real-time and persistent effects of ROM restriction on forelimb trajectories during stepping. However, only spinal subjects showed persistent effects of ROM restriction (on hindlimb activity). This corresponds with previous research showing that animals can adapt to and show persistent changes in behavior following a spinal cord transection (Viala et al., 1986; Grau, 2001; Wolpaw, 2006, 2007; Brumley and Robinson, 2010).

### Effects of ROM restriction on interlimb coordination (stepping)

ROM restriction influenced fore- and hindlimb step frequency during the period of restriction. While a decrease in synchronized forelimb stepping corroborates a previous study in 1-day old intact rats (Brumley et al., 2012), ROM-restricted spinal pups in the current study also exhibited a reduction in alternated hindlimb stepping during the restriction period. As shown in Figure 4A, non-ROM-restricted spinal pups showed a 3-fold increase in alternated hindlimb steps following quipazine treatment. However, ROM restriction suppressed this effect. Because ROM restriction suppressed hindlimb stepping starting 10-min after restriction, but not within the first 5-min, perhaps the initial increase in stepping gave spinal subjects more “trials” with the perturbation and hence more sensory feedback to respond to (compared to shams). Thus, additional steps may have helped facilitate responsiveness to ROM restriction. Another possibility is that quipazine had different neuromodulatory effects on sensory responsiveness in sham vs. spinal subjects. For example, Chopek et al. (2013) showed that quipazine preferentially increases the monosynaptic reflex of flexor nerves in spinal-transected compared to spinal-intact adult rats. Thus, sensory processing of the perturbation was likely different in the two surgery conditions.

Despite subjects showing real-time effects, no persistent effects of ROM restriction (following removal of the perturbation) were seen on step frequency. One possibility for lack of persistent changes may be a result of limited exposure to ROM restriction. Previous studies of interlimb motor learning after spinal transection, such as conjugate limb yoking studies, typically expose subjects to the sensory perturbation (interlimb yoke) for around 30 min (Brumley and Robinson, 2010). Thus, extending the exposure time in the current study may have allowed persistent effects to emerge. However, given that quipazine markedly increases motor activity in neonatal rats, we assumed that more motor activity might provide more sensory feedback and thus a shorter exposure time might be sufficient to induce persistent effects. Another possibility is that persistent effects are age-dependent. In a study with human infants, persistent adaptations to trip training on a treadmill were not reliably seen until about 9 months of age, despite infants showing real-time changes (Pang et al., 2003). Given research that equates P10 rats with 9–10 month old human infants in terms of locomotor development (Vinay et al., 2005), but only the late-term human fetus in terms of brain development (Clancy et al., 2001), our subjects may be too immature to show reliable lasting effects. However, studies examining interlimb yoke training have found persistent changes in motor coordination in rat fetuses and newborns (Robinson, 2005; Robinson et al., 2008; Brumley and Robinson, 2010), suggesting that persistent interlimb changes should be possible. Therefore, future studies should examine key features necessary to produce reliable, persistent coordinative changes following a spinal cord transection.

It is curious that ROM restriction leads to a decrease in step frequency, given the large body of research showing that sensory feedback often facilitates locomotor recovery after SCI (e.g., Carhart et al., 2004; Teulier et al., 2009). However, as observed in the current study, subjects that received ROM restriction changed their intralimb trajectories (discussed below). This alteration in intralimb coordination may have compromised the ability of the pup to maintain interlimb coordination during stepping.

### Effects of ROM restriction on intralimb coordination (limb trajectories)

During ROM restriction, the perturbation blocked part of the center and distal limb trajectory areas. Consequently, ROM-restricted pups produced fewer limb movements in these areas. To adapt, pups tended to move their forelimbs mostly to the front area during ROM restriction. For the hindlimbs, pups moved more-or-less equally to front and back areas, while avoiding the center during restriction. Further, during restriction, intralimb adaptations in spinal subjects appeared to be more drastic in the hindlimbs compared to forelimbs (i.e., much less time was spent in center and distal areas). As noted above, these intralimb adaptations may have interfered with interlimb coordination. One reason that the limbs may have adapted by mainly moving to the front of the subject rather than behind, may be related to the differential effect of serotonergic stimulation on flexor and extensor motor output. Quipazine has been shown to preferentially increase flexor motor output in spinal rats (Chopek et al., 2013), and likewise a lack of serotonin increases limb extension (Pflieger et al., 2002). In the current study, the limbs could be relatively flexed and remain in the front of the animal, but to move to the back the limbs would require significant extensor activity. Thus, perhaps the movement of the limbs mainly to the front trajectory area is indicative of a stronger effect on flexor activity rather than extensor activity, following treatment with a 5-HT receptor agonist.

Persistent effects of ROM restriction were seen immediately after the perturbation was removed in both fore- and hindlimbs. Persistent forelimb effects lasted for a longer period of time in spinal pups. One possibility for longer lasting persistent effects in spinal pups may be due to the isolation of the forelimbs from the hindlimbs. Blocking ascending input from the caudal spinal cord, including propriospinal neurons, may make sensory feedback from the forelimbs more salient since there is less input into the rostral cord (compared to shams). Blocking caudal input may be especially important given research suggesting that quipazine-induced hindlimb activity may help drive the forelimbs (McEwen et al., 1997; Brumley and Robinson, 2005). Another possibility is changes in somatosensory cortex processing. Hindlimb areas within the cortex failed to respond to hindlimb stimulation but instead responded to forelimb stimulation, in adult rats transected as neonates (Kao et al., 2011). Thus, feedback from the forelimbs may be activating more brain regions in spinal animals and facilitate lasting intralimb adaptations. Alternatively, given that spinal pups in the current study had been living with a spinal cord transection for 9 post-operative days, they may have adopted novel posture and movement strategies during this period. The duration of such intralimb adaptations, or whether or not such changes might persist from one test session to the next, remains to be determined.

Besides changes in intralimb coordination for ROM-restricted subjects, quipazine also altered intralimb coordination for pups in both surgery conditions. Specifically, pups showed a decrease in the center and distal areas following treatment with quipazine. This is likely the result of quipazine increasing the amount of stepping behavior, as stepping involves both limb flexion and extension, with the limbs typically showing locomotor-like swing and stance limb excursions. Thus, it is not surprising that pups treated with quipazine (but not ROM restriction) utilized their movement space differently from baseline.

### Quipazine-induced stepping in spinal subjects

As mentioned above, P10 rats that received a low thoracic spinal cord transection on P1 showed three times as many hindlimb steps following treatment with quipazine, compared to shams. Researchers looking at what has been termed “hindlimb supersensitivity” have found changes in the spinal cord following a transection that may help account for this apparent sensitivity to stimulation at 5-HT receptors. For example, in chronic transected rats the concentration of 5-HT_2_ receptors has been shown to increase 3 to 5-fold throughout motor neuronal somata and dendrite regions, within the caudal cord (Kong et al., 2010). A study that examined the time course of changes in 5-HT receptors after a complete spinal transection reported an increase in 5-HT_2_ receptors beginning 24 h after surgery (Kong et al., 2011). Another study examined changes in motor neuron excitability and found that small doses (10–50 μM) of a 5-HT_2_ agonist produced cell depolarization, increased input resistance, and large persistent inward currents in adult spinal rats (Harvey et al., 2006). Specifically, 5-HT_2A_ receptor stimulation has been shown to restore hyperpolarizing inhibition in spinal motor neurons via upregulating activity and expression of the K-Cl cotransporter KCC2 in the isolated neonatal rat spinal cord *in vitro* (Bos et al., 2013). This permits endogenous reciprocal inhibition necessary for maintaining left-right alternation seen during locomotion, which can be activated in the isolated spinal cord *in vitro* and *in vivo* with 5-HT_2A_receptors (Norreel et al., 2003; Pearlstein et al., 2005). Thus, it is possible that spinal pups in the current study experienced an up-regulation of 5-HT_2_ receptors, motor neuronal hyperexcitability responses to quipazine, and enhanced reciprocal inhibition, and therefore the effects of quipazine (a 5-HT_2A_receptor agonist) were much more pronounced in these pups compared to shams. Further support for the role of serotonergic stimulation in regulating lumbar networks during early development comes from studies that have demonstrated impaired locomotor coordination, posture, and motor neuron excitability following serotonin depletion (Myoga et al., 1995; Nakajima et al., 1998; Pflieger et al., 2002).

An interesting finding in the current study was the occurrence of alternated forelimb stepping in spinal pups treated with quipazine (although the amount of forelimb stepping was much lower than hindlimb stepping). Previous studies looking at quipazine-induced stepping in perinatal rats have reported robust hindlimb stepping, but very little forelimb stepping after a mid-thoracic spinal transection (McEwen et al., 1997; Brumley and Robinson, 2005). However, the current study differs from these studies in a couple of important ways, which may help to explain this difference in forelimb behavior. First, the previous studies performed behavioral testing within 24 h after spinal transection, whereas in the current study pups were allotted 9 days after surgery to recover before testing. Therefore, longer recovery time may have allowed pups to fully recover from any spinal shock rostral to the injury site. Second, the current study used a low thoracic spinal cord transection, whereas the previous studies used mid-thoracic transections. Thus, it is possible that the neural circuitry for forelimb stepping extends into the high thoracic area, and therefore a mid-thoracic transection interferes with or damages forelimb stepping circuitry. However, with a low thoracic transection, perhaps we missed that circuitry altogether, and therefore animals could easily show stepping behavior in the forelimbs.

## Conclusions

Findings from this study suggest that the immature, isolated spinal cord modulates inter- and intralimb coordination in response to sensory feedback during locomotor activity induced by serotonergic stimulation. Furthermore, we found that the spinal cord is able to support persistent behavioral changes after exposure to a sensorimotor perturbation. A number of studies have shown the importance of pairing sensory input with spinal cord circuitry activation. In these studies, the addition of 5-HT receptor stimulation is often shown to modulate changes in recovery. For example, subjects given motor training typically do not recover to the same extent as subjects given motor training plus treatment with a 5-HT agonist. Interestingly, a study that examined mice and rats with a chronic spinal cord contusion found that increases in behavioral recovery were correlated with an increase in 5-HT receptor expression in the spinal cord (Wang et al., 2011). Thus, 5-HT stimulation is not just a tool to activate locomotor circuits but is likely part of a dynamic system involved in the production, modulation, and recovery of functional movement. By understanding how 5-HT modulates locomotor behavior and how sensory feedback and supraspinal input changes 5-HT expression, we can gain a more accurate picture of how to tailor therapies toward better recovery. Such therapeutic strategies can already be seen in studies with humans where sensory feedback (i.e., treadmill training) is paired with activation of local spinal circuits (e.g., epidural stimulation) (Carhart et al., 2004).

### Conflict of interest statement

The authors declare that the research was conducted in the absence of any commercial or financial relationships that could be construed as a potential conflict of interest.
